# Effects of the *scid* mutation on X-ray-induced deletions in the brain and spleen of *gpt* delta mice

**DOI:** 10.1186/s41021-020-00158-y

**Published:** 2020-05-24

**Authors:** Kenichi Masumura, Fumio Yatagai, Masako Ochiai, Hitoshi Nakagama, Takehiko Nohmi

**Affiliations:** 1 Division of Genetics and Mutagenesis, National Institute of Health Sciences, 3-25-26 Tonomachi, Kawasaki-ku, Kawasaki-shi, Kanagawa 210-9501 Japan; 2 Center for Sustainable Resource Science, The Institute of Physical and Chemical Research, 2-1 Hirosawa, Wako-shi, Saitama, 351-0198 Japan; 3 Biochemistry Division, National Cancer Center Research Institute, 5-1-1 Tsukiji, Chuo-ku, Tokyo, 104-0045 Japan; 4 Present Address: Department of Animal Experimentation, National Cancer Center Research Institute, 5-1-1 Tsukiji, Chuo-ku, Tokyo, 104-0045 Japan; 5 Present Address: National Cancer Center, 5-1-1 Tsukiji, Chuo-ku, Tokyo, 104-0045 Japan

**Keywords:** DNA-PKcs, *scid* mice, Non-homologous end-joining, Spi^−^ assay, Deletion, X-irradiation

## Abstract

**Background:**

DNA-dependent protein kinase (DNA-PK), consisting of a Ku heterodimer (Ku70/80) and a large catalytic subunit (DNA-PKcs), plays an important role in the repair of DNA double-strand breaks via non-homologous end-joining (NHEJ) in mammalian cells. Severe combined immunodeficient (*scid*) mice carry a mutation in the gene encoding DNA-PKcs and are sensitive to ionizing radiation. To examine the roles of DNA-PKcs in the generation of deletion mutations in vivo, we crossed *scid* mice with *gpt* delta transgenic mice for detecting mutations.

**Results:**

The *scid* and wild-type (WT) *gpt* delta transgenic mice were irradiated with a single X-ray dose of 10 Gy, and Spi^−^ mutant frequencies (MFs) were determined in the brain and spleen 2 days after irradiation. Irradiation with X-rays significantly enhanced Spi^−^ MF in both organs in the *scid* and WT mice. The MFs in the brain of irradiated *scid* mice were significantly lower than those in WT mice, i.e., 2.9 ± 1.0 × 10^− 6^ versus 5.0 ± 1.1 × 10^− 6^ (*P* < 0.001), respectively. In the spleen, however, both mouse strains exhibited similar MFs, i.e., 4.1 ± 1.8 × 10^− 6^ versus 4.8 ± 1.4 × 10^− 6^. Unirradiated *scid* and WT mice did not exhibit significant differences in MFs in either organ.

**Conclusions:**

DNA-PKcs is unessential for the induction of deletion mutations in the spleen, while it plays a role in this in the brain. Therefore, the contribution of DNA-PKcs to NHEJ may be organ-specific.

## Introduction

The repair of DNA double-strand breaks (DSBs) is critical for the maintenance of genomic integrity. In mammalian cells, DSBs are repaired by the homologous recombination and/or the nonhomologous end-joining (NHEJ) pathways [1–3]. However, DSBs induced by ionizing radiation (IR) are mainly repaired through NHEJ pathway. This is particularly true in non-dividing cells and in G1 cells due to the absence of sister chromatids, the preferred substrate for homologous recombination. Although defects in NHEJ result in genomic instability and cancer predisposition, NHEJ often leads to deletion mutations, with or without short length insertions, when DNA ends can’t be directly ligated [4]. IR and chemical treatments usually induce modified DSBs with ends that are incompatible for direct ligation. Therefore, NHEJ can be regarded as a double-edged sword; while it prevents cell death and gross chromosome rearrangements, it often induces deletions and insertions during ligation of incompatible ends.

DNA-dependent protein kinase (DNA-PK) consists of three components, the catalytic subunit DNA-PKcs and the heterodimeric Ku70 and Ku80 proteins, and is involved in NHEJ of DNA DSBs and V(D)J recombination [5–9]. In general, when DNA DSBs are induced, Ku70/80 proteins bind to the ends and interact with other proteins including DNA PKcs and Artemis for end-resection, DNA polymerase μ and λ for addition of nucleotides, and the DNA ligase IV complex for ligation of the ends. However, different proteins are recruited to the site of DNA damage to participate in the repair of DSBs each time, depending on the end configurations i.e., blunt ends, 3′- or 5′-overhands or ends containing modified bases [10].

The *scid* (severe combined immune-deficiency) mice bear a naturally occurring mutation in the DNA-PKcs gene that results in an 83-amino acid truncation of the C-terminal end [11–14]. These mice, and cells derived from them, are hypersensitive to IR, display defects in joining of IR-induced DNA DSBs [12, 15], and are defective in coding joint formation during V(D)J recombination [16, 17]. However, *scid* cells can form signal joints in V(D)J recombination [18], which involve the joining of the ends created after the excision of intervening DNA during V(D)J recombination and the formation of circular DNA molecules. In contrast, Ku-deficient mice and embryonic stem cells exhibit defects in both coding and signal joint formation [19–22]. Therefore, it appears that DNA-PKcs is only needed to resolve a subset of DSBs and that NHEJ may proceed in a DNA-PKcs-independent, as well as DNA-PKcs-dependent, manner.

We previously developed the *gpt* delta transgenic mouse for the detection of mutations in vivo [23–25]. In *gpt* delta mice, about 80 copies of lambda EG10 DNA, which carries *red* and *gam* genes, are integrated into each chromosome 17 in a C57BL/6 J background [26, 27]. A feature of the mutation assay is its ability to efficiently detect certain types of deletions by Spi^−^ (sensitive to P2 interference) selection, as well as point mutations, i.e., base substitutions and frameshifts, by 6-thioguanine selection [28, 29]. Spi^−^ selection takes advantage of the restricted growth of the lambda phage in P2 lysogens [30]. Only mutant lambda phages that are deficient in the functions of both *red* and *gam* genes can grow well in P2 lysogens and display the Spi^−^ phenotype. Simultaneous inactivation of the two adjacent genes is usually induced by a deletion in the region, or frameshifts that interfere with the translation of both genes. Spi^−^ selection detects deletions ranging from single nucleotide (− 1) frameshifts to 10 kb in size [29]. In this procedure, the lambda EG10 is rescued from the mouse genome by in vitro packaging reactions, and P2 lysogens are infected with the rescued phages to identify Spi^−^ plaque. We have demonstrated that the Spi^−^ mutant frequency (MF) is substantially increased by IR and chemical treatments, and suggested that NHEJ repair plays an important role in the induction of Spi^−^ deletion mutants [28, 31, 32].

Since NHEJ may proceed in DNA PKcs-dependent and independent manners, it may be possible that DNA PKcs plays a significant role in deletion formation in one organ while it plays only a negligible role in another one. It is reported that MF of unirradiated *Ku80*^−/−^ mice is higher than that of WT mice in the spleen while the MFs are similar between two strains of mice in the liver [33]. To examine the possible variation of the roles of DNA PKcs in deletion mutations in mammalian organs, we crossed *scid* mice with *gpt* delta mice (hereafter, the offspring from this cross will be referred to as *scid* mice, and *gpt* delta mice as wild-type (WT) mice). We irradiated *scid* and WT mice with X-rays and compared Spi^−^ MFs in the brain and spleen, which are representative organs with quiescent and proliferating cells, respectively. The results indicated that X-ray irradiation significantly induced deletion mutations in both organs of *scid* and WT mice. Although *scid* mice exhibited significantly lower MFs than WT mice in the brain, both mouse strains exhibited similar MFs in the spleen. Possible mechanisms of DNA-PKcs-independent NHEJ of DNA DSBs are discussed.

## Materials and methods

### Treatment of animals

C.B-17 *scid* mice, maintained in CLEA Japan, were crossed with C57BL6/J lambda EG10-homozygous *gpt* delta mice [26]. The heterozygous F1 mice were mated with the same offspring carrying the *scid* mutation and lambda EG10 transgene, which resulted in F2 mice. The *scid* genotype of each experimental animal was confirmed by PCR, according to a previously reported method [13]. The existence of the transgene was also confirmed using a previously reported method [26]. Eleven- to twelve-week-old WT (*wt/wt*) and *scid* (*scid/scid*) mice were whole-body irradiated with X-rays; a total dose of 10 Gy. X ray-irradiation (200 kVp, Softex-Rigaku) was delivered at a dose rate of 1 Gy per min. Each group consisted of a total of 6–10 male and female mice. The mice were sacrificed 2 days following irradiation. The brain and spleen were collected and quickly frozen in liquid nitrogen and stored at –80 °C. Genomic DNA was extracted from the organs using the phenol/chloroform method and lambda EG10 phages were rescued using Transpack^R^ Packaging Extract (Agilent Technology, Japan) as described previously [24].

### Spi^−^ mutation assay

The Spi^−^ mutation assay was performed as described previously [24]. The rescued phages were used to infect *E. coli* XL1-Blue MRA (P2) cells. The infected cells were mixed with molten soft agar, poured on lambda-trypticase agar plates, and incubated at 37 °C. The Spi^−^ candidate plaques detected on the plates were suspended in 50 μL of SM buffer. The suspension was spotted on the plate where the XL1-Blue MRA (P2) cells were spread. The plates were incubated at 37 °C; the mutants that produced clear spots were counted as confirmed Spi^−^ mutants. The rescued phages were also used to infect *E. coli* XL1-Blue MRA cells to determine the number of rescued phages. The Spi^−^ MF was calculated as described previously [24]. Phage lysates of the Spi^−^ mutants were used as templates for PCR analysis. The PCR primers were:

primer 001 (5′-CTCTCCTTTGATGCGAATGCCAGC-3′),

primer 002 (5′-GGAGTAATTATGCGGAACAGAATCATGC-3′),

primer 005 (5′-CGTGGTCTGAGTGTGTTACAGAGG-3′),

primer 006 (5′-GTTATGCGTTGTTCCATACAACCTCC-3′) and.

primer 012 (5′-CGGTCGAGGGACCTAATAACTTCG-3′).

The appropriate primers for DNA sequencing were selected based on the results of PCR analysis. The sequencing primers have been described previously [34–36]. The entire sequence of lambda EG10 is available at http://www.nihs.go.jp/dgm/dgm3/eg10v20.txt. DNA sequencing was performed with BigDye™ Terminator Cycle Sequencing Kit (Applied Biosystems, Foster City, CA) and ABI PRISM™ 310 Genetic Analyzer (Applied Biosystems).

### Statistical analysis

All data were expressed as mean ± standard deviation (SD). A Tukey-test was used to determine if the differences between two groups were statistically significant. A *P* value of < 0.05 was considered statistically significant.

## Results

### Spi^−^MFs in the brain and spleen of X-ray-irradiated mice

**Fig. 1:**
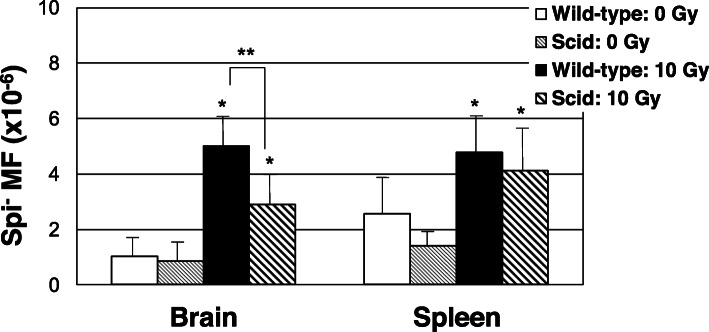
The Spi^−^ MFs in the brain and the spleen of WT and *sicd* mice with or without X-ray-irradiation. Error bars mean ± SD of the MFs in each group; *n* = 10 for WT (0 Gy), *n* = 8 for WT (10 Gy), *n* = 6 for *scid* (0 Gy) and *n* = 7 for *scid* (10 Gy). * *P* < 0.01, relative to the unirradiated groups. ** *P* < 0.01, relative to the WT mice. A Tukey-test was used to determine if the differences between two groups were statistically significant

WT and *scid* mice were exposed to X-ray irradiation at a dose of 10 Gy and Spi^−^ MFs were determined in the brain and spleen (Fig. 1, Supplementary Table 1 and 2). X-ray irradiation significantly enhanced Spi^−^ MFs in both organs in WT and *scid* mice. The MFs in the brain of *scid* mice were significantly lower than those of WT mice, i.e., 2.9 ± 1.0 × 10^− 6^ versus 5.0 ± 1.1 × 10^− 6^, *P* < 0.001. In the spleen, however, both mouse strains exhibited similar MFs after irradiation (4.1 ± 1.8 × 10^− 6^ vs 4.8 ± 1.4 × 10^− 6^, *P* = 0.77). Unirradiated *scid* and WT mice did not exhibit significant differences in MFs in either organ (0.85 ± 0.67 × 10^− 6^ vs 1.0 ± 0.64 × 10^− 6^ in the brain and 1.4 ± 0.31 × 10^− 6^ vs 2.6 ± 1.3 × 10^− 6^ in the spleen).

### Deletion mutation spectra in the brain of WT and *scid* mice

Since MFs in the brain were significantly lower in *scid* mice than in WT mice, we were interested in whether the spectra of deletions were different in the two strains of mice. Therefore, we sequenced the Spi^−^ mutants recovered from the brain of X-ray-irradiated and unirradiated WT and *scid* mice (Tables 1 and 2). The specific MFs of each type of deletion were calculated by multiplying the MF by the ratio of the number of each class of mutations to the total number of deletion mutations.

**Table 1:** Spi^−^ mutation spectra in the brain of X-ray-irradiated WT and the *scid* mice

	WT 0 Gy			WT 10 Gy			*scid* 0 Gy			*scid* 10 Gy		
	No.	%	MFx10^−6^	No.	%	MFx10^−6^	No.	%	MFx10^−6^	No.	%	MFx10^−6^
1 bp deletion												
In run	18	72	**0.74**	30	42	**2.08**	6	75	0.64	40	42	**1.22**
Other	2	8	**0.08**	6	8	**0.42**	0	0	0.00	10	11	**0.30**
> 2 bp deletion												
With microhomology	0	0	**0.00**	21	29	**1.46**	0	0	0.00	25	26	**0.76**
Without microhomology	3	12	**0.12**	12	17	**0.83**	1	13	0.11	11	12	**0.33**
Insertion	0	0	**0.00**	2	3	**0.14**	0	0	0.00	4	4	**0.12**
Insertion	2	8	**0.08**	0	0	**0.00**	0	0	0.00	5	5	**0.15**
Complex	0	0	**0.00**	1	1	**0.07**	1	13	0.11	0	0	**0.00**
Total	25	100	**1.03**	72	100	**5.00**	8	100	0.85	95	100	**2.89**

Specific MFs were calculated by multiplying the total MF by the ratio of each class of mutants

In unirradiated WT mice, total 26 mutants (suppl. Table 1) were obtained. They were sequenced and 25 deletion mutations (Table 1) were identified. In 10 Gy-irradiated WT mice, total 172 mutants were obtained. Eighty seven mutants were sequenced and 72 deletion mutations were identified. In unirradiated *scid* mice, total 9 mutants were obtained. All were sequenced and 8 deletion mutations were identified. In 10 Gy-irradiated *scid* mice, total 67 + 89 mutants were obtained. One hundred and twenty five were sequenced and 95 deletion mutations were identified. In 10 Gy-irradiated *scid* mice, 89 mutants were obtained by additional plating for sequencing analysis, but they were not used for calculation of MF because no additional plating was conducted for control groups

**Table 2:** Summary of Spi^−^ mutations in the brain of X-ray-irradiated WT and *scid* mice

Types of deletions	Position	Position	Sequence	Sequence	No. of mutants			
	in *gam*	in lambda EG10	Change ^a^	at junction ^b,c^	WT	*scid*	WT	*scid*
					0 Gy	0 Gy	10 Gy	10 Gy
One base pair deletions								
In run sequences								
	141–142		GG→G				1	
	188–190		CCC→CC					1
	199–201		AAA→AA				1	
	227–231		AAAAA→AAAA		5^d^	1	5^d^	11^d^
	238–241		CCCC→CCC		2^d^	1	1	2^d^
	286–289		GGGG→GGG		5^d^	1	3^d^	12^d^
	290–291		CC→C				1	1
	295–300		AAAAAA→AAAAA		6^d^	3^d^	15^d^	9^d^
	316–318		TTT→TT				1	
	334–336		TTT→TT					2
	377–378		CC→C					1
	380–381		TT→T				1	
	387–388		CC→C				1	
	390–391		CC→C					1
Other 1 bp deletion								
	131		ttAtt→tttt					1
	175		cacTac→cacac					1
	183		caGct→cact					1
	203		gAgg→ggg					1
	218		agAcg→agcg		1			
	236		ccTgc→ccgc					1
	268		tcGat→tcat				1	
	276		tttG→ttt				1	
	277		ttgCaac→ttgaac					2^d^
	285		Cgggg→gggg				1	
	294		Caaaaaa→aaaaaa		1			
	301		aaaaaaT→aaaaaa					1
	320		tttgAt→tttgt					1
	328		tGtt→ttt					1
	332		gAg→gg				1	
	341		ggAg→ggg				1	
	392		ccAgg→ccgg				1	
> 2 bp deletions								
Deleted sizes (bp)								
2	153 → 156			tgag tcag			1	
2	251 → 254			tgtt aatc				1
2	349 → 352			atgg gaac			1	
3	355 → 359			gaac tccg				1
4	182–184 → 187–189			agca**GC**ccgt			1	
4	222 → 227			cgac aaaa				1
4	239–240 → 244–245			tgcc**C**acct				1
4	247–249 → 251–253			cacc**T**gaat			1	
4	295–300			cagc**AA**tcca				1
4	304 → 309			tcca ccgt				1
5	300–301 → 306–307			aaaa**T**accc				2
7	147–149 → 155–157			tcgt**CT**caga				1
7	349–351 → 357–359			atgg**CA**tccg			1	
8	175 → 184			cact ctcg			1	
10	196–198 → 207–209			gaag**AG**aact			1	
10	323 → 334			atga tttc			1	
10	335 → 346			agtt atgg			1	
10	375–379 → 386–390			tgaa**AC**cacc			1	
12	221–222 → 234–235			acga**C**ctgc				1
12	313 → 326			cgtg atgt			1	
13	376–379 → 389–392			gaaa**CC**Aggtt			1	
14	165–167 → 180–182			ctgg**G**cagc			1	
17	154–155 → 172–173			gagg**C**acta			1	
17	239–240 → 257–258			ctgc**C**gcta				1
17	251 → 269			tgtt atca				1
19	212–215 → 232–235			aact**GGC**ctgc				1
22	288–289 → 311–312			cggg**G**tgcg				3
26	268 → 285			atcg cggg				2^d^
28	307 → 336			atta tcag			1	
28	338 → 367			ttca atgg		1		
32	189–190 → 222–223			cgcc**C**atgg			1	
34	346–348 → 381–383			cgca**TG**ctca			1	
41	380 → 422			ccat aatg				1
49	206 → 257 (1 bp ins.)			aggc ***C*** cgct			1	
67	246–247 → 314–315			gcac**C**gttt				1
76	189 → 266			cgcc tcga			1	
86	252–253 → 339–340			gttt**G**gagc			1	
107	91–92 → 199–200			cgat**A**aaga			1	
124	208 → 333			gcag gttt				1
127	273–276 → 401–404			tcat**TTG**attc				1
151		24,867–24,870 → 25,019–25,022		cgac**ACG**cacg			1	
432		24,828–24,830 → 25,261–25,263		gagt**GG**gctg				1
449		24,683–24,684 → 25,133–25,134		ccca**C**tttc				1
596		24,446–24,450 → 25,043–25,047		ata**TGGC**cccg				1
654		24,719 → 25,374		aagg tcgc				1
1248		24,524–24,536 → 25,767–25,779		aatg**GTTCGCGGCGGC**gtg				2
1436		24,563 → 26,000		caga cagt		1		
1557		24,222 → 25,802 (22 bps ins.)		tgtc ***CATTCAAAACACACCACCAAAG*** ctcc				1
1616		23,938–25,555 (2 bps ins.)		aaac ***AG*** gcct			1	
1856		23,960–23,961 → 25,817–25,818		gaag**T**tggt			1	
1874		24,161–24,162 → 26,009–26,010		tgtt**T**gctg			1	
2124		23,141 → 25,266 (1 bp ins.)		tcgg ***A*** gatt				2
2388		23,000–23,004 → 25,389–25,393		tgct**GCGA**tag				1
2388		24,000–24,002 → 28,232–28,234		ggg**GT**gtca				1
2441		24,034–24,035 → 26,476–26,477		cggt**G**ccag				1
2842		24,247–24,248 → 27,090–27,091		agc**G**ccga				1
3628		25,062 → 28,691		aaa ctg			1	
3701		24,560 → 28,262		gatg gcac				1
3707		21,458–21,459 → 25,166–25,167		att**G**cgcc				1
3979		22,200–22,204 → 26,180–26,184		ccag**TTTA**tttt			2	
4144		22,585–22,586 → 26,730–26,731		cgtt**C**tgcc				1
4689		23,611–23,612 → 28,301–28,302		agtt**G**cgcg			1	
4698		24,423–24,424 → 29,122–29,123		gaa**G**tgcc				1
4841		21,691–21,692 → 26,533–26,534		agac**A**tcat			1	
5037		21,355 → 26,393		ctct agaa			1	
5251		19,712–19,713 → 24,964–24,965		cacc**A**ccat			1	
5422		22,340 → 27,760 (3 bps ins.)		cgcc ***TTT*** caca				1
5562		19,997 → 25,560		atag gatt	1			
5727		19,335 → 25,063		tggc tgat			1	
6900		24,036 → 30,937		gtga gatc			1	
7303		23,917 → 31,221		cttc tcgt	1			
9030		21,854–21,857 → 30,885–30,888		gagt**ACG**cttt			1	
Insertions								
+ 1	227–231		AAAAA→AAAAAA		1			
+ 1	227–231		AAAAA→AAAAAA					1
+ 1	295–300		AAAAAA→AAAAAAA					4^d^
+ 1	356		aaca→aacTa		1			
Complex								
N.D.		23,999 → 24,381, 23,997–23,996 → 27,762–27,763				1		
N.D.		21,108–21,109 → 13–14					1	
					25	8	72	95

^a^ Capital letters are deleted or Inserted bases

^b^ Bold and underlined bases denote homologous sequences of deletion junctions

^c^ Bold and italic bases denote inserted sequences at deletion junctions

^d^ The mutations were independently observed from more than two different mice

In WT mice, the frequency of 1 base pair (bp)-deletions increased 3.0-fold by irradiation (0.82 to 2.50 × 10^− 6^), while the frequency of deletions of more than 2 bps in size, increased 20.3-fold (0.12 to 2.43 × 10^− 6^). Among the sequenced 35 deletions of more than 2 bps in size, 37% (13/35) were deletions of more than 1 kb and 60% (21/35) had microhomologous sequences of 1 to 4 bps at the junction. The average length of microhomology was 1.8 bp. Six % (2/35) had an inserted base at the junctions.

In *scid* mice, the frequency of 1 bp-deletions increased 2.4-fold by irradiation (0.64 to 1.52 × 10^− 6^), while the frequency of deletions of more than 2 bps in size, increased 11.1-fold (0.11 to 1.22 × 10^− 6^). Among the sequenced 40 deletions of more than 2 bps in size, 35% (14/40) were more than 1 kb and 63% (25/40) had microhomologous sequences at the junction. With the exception of one mutation that had a 12-bp microhomology, the average length of microhomology was 1.7 bp. Five mutations containing a 1 bp-insertion, not accompanied by deletions, were observed in irradiated *scid* mice, whereas no such mutations were observed in irradiated WT mice.

There was no significant difference in the mutation spectra between unirradiated WT and *scid* mice. One bp-deletions in the repetitive sequences were the most dominant type of mutation. Three hotspots were observed in unirradiated mice: 1) AAAAA to AAAA at position 227–231, 2) GGGG to GGG at position 286–289 and 3) AAAAAA to AAAAA at position 295–300 in the *gam* gene.

## Discussion

Although it is well known that the *scid* mice are severely sensitive to killing effects of irradiation, little is known about the roles of DNA PKcs in irradiation-induced deletion mutations in various organs of mice. In this study, the WT and *scid* mice were irradiated with X-rays and deletion mutations were analyzed in the brain and the spleen. In the X-ray-irradiated WT mice, the Spi^−^ MFs in the brain and spleen were significantly higher than those of unirradiated mice. Sequencing analysis of the Spi^−^ mutants in the brain showed that X-ray irradiation preferentially induced large deletions of up to 10 kbps (Table 1). Specific MFs of deletions of more than 2 bps in size increased 20.3-fold upon irradiation, in contrast to the MFs of deletions of 1 bp that increased 3.0-fold. Among the sequenced 35 deletions of more than 2 bps in size, 60% (21/35) had microhomologous sequences of 1–4 bps at the deleted junction (Table 2). These data confirmed that deletions of more than 2 bps in size in this study are largely generated through NHEJ of DNA DSBs [3, 29].

In irradiated *scid* mice, the Spi^−^ MFs were significantly increased 3.2- and 2.9-fold in the brain and spleen, respectively, compared with those of unirradiated *scid* mice. In the brain, the specific MF of deletions of more than 2 bps in size increased 11-fold by irradiation, in contrast to the MF of 1 bp-deletions that increased 1.9-fold (Table 1). Sequencing analysis of the Spi^−^ mutants showed that 63% (25/40) of the deletions of more than 2 bps in size had microhomologous sequences of 1–12 bps at the deleted junction. These characteristics of the X-ray- induced deletions in *scid* mice were similar to those of WT mice, suggesting that X-ray-induced DSBs are repaired by NHEJ even without DNA-PKcs. It is possible, however, that the defective protein encoded by the murine *scid* allele retains enough residual function to support NHEJ. Bogue et al. examined V(D)J recombination in DNA-PKcs-deficient SLIP mice and found that the effects of this mutation on coding and signal joint formation are identical to the effects of the *scid* mutation [18]. These data are incompatible with the notion that signal joint formation in *scid* mice results from residual DNA-PKcs function and support the idea that DNA-PKcs is not an essential factor for NHEJ in mice. The analysis of DNA-PKcs knock-out mice also supports this idea [37]. Hence, we suggest that DNA-PKcs-independent NHEJ is responsible for deletions associated with X-ray exposure in the spleen and, in part, in the brain.

How can NHEJ proceed without DNA-PKcs in the brain and spleen? DNA-PKcs interacts with the C-terminal part of Ku80, a component of DNA-PK. When Ku binds to DNA ends, the interaction with DNA PKcs increases substantially, which leads to autophosphorylation of DNA PKcs and activation of the endonuclease activities of Artemis [38]. This endonuclease appears to play a role in the removal of 5′- and 3′-overhangs in DNA ends, which seems to be a necessary step for the efficient ligation of broken DNA ends. It is estimated, however, that more than half of IR-induced DSBs are repaired even without the activities of Artemis [39, 40]. This suggests that nucleases other than Artemis, such as Apratoxin and PNKP-like factor (APLF), flap endonuclease (FEN1), DNA replication helicase/nuclease 2 (DNA 2) and exonuclease 1, may play roles in the resection of broken DNA with incompatible DNA ends [3]. We speculate, therefore, that DNA PKcs-independent nucleases may play roles in NHEJ in the brain and spleen while the canonical DNA PKcs-dependent Artemis is involved in NHEJ in the brain. Obviously, further work is needed to reveal exact mechanisms by which NHEJ proceeds without DNA PKcs in the organs.

Unirradiated *scid* and WT mice did not exhibit significant differences in MFs in the brain and spleen (Fig. 1). In addition, the spectra of deletions were similar between the two strains of mice, where 1 bp-deletions in the repetitive sequences were the most dominant. These deletions, however, are mostly generated by slippage of DNA polymerases during DNA replication and not during DNA repair of DSBs [29]. It is reported that spontaneous *lacI* MFs were similar between *scid* and WT mice in the brain, spleen and liver [41]. Spontaneous MF of expanded simple tandem repeat (ESTR) in male germline was higher in the *scid* mice than in the WT mice although the frequency was not enhanced by irradiation [42].

Lee et al. report that DNA-PK activity fluctuates in a cell cycle-dependent manner, and propose a model in which two illegitimate recombinational repair pathways exist in mammals, one of them being DNA-PK dependent and restricted to the G1/early S phase and the other being DNA-PK independent and restricted to the late S/G2 phase of the cell cycle [43]. They showed that the DNA DSB repair activity in the *scid* pre-B cells was greatly reduced during the G1/early S phase resulting in increased X-ray hypersensitivity but was indistinguishable from that in WT cells during the late S and G2 phases. In the spleen, in which cell division is active, DNA-PK independent repair may work well during the late S and G2 phases and neutralize the effect of the *scid* mutation. In the brain, in which cell division is inactive, DNA-PK dependent end-joining may play an important role in DSB repair and, in this context, the *scid* mutation may lead to a reduced activity of DSB repair and the induction of deletion mutations. In the mutation spectra of Spi^−^ mutations recovered from the brain of the irradiated *scid* mice, there were 4 mutations having insertion sequences at the deleted junctions and 5 mutations having + 1 insertions. This was observed in 9.5% (9/95) of the analyzed samples (Table 2). In the irradiated WT mice, there were 2 mutations having insertion sequences at the deleted junctions and no mutations having + 1 insertions. This was observed in 2.8% (2/72) of the analyzed samples. The higher frequency of mutations with insertions that was observed in the irradiated *scid* mice raises the possibility that DNA-PKcs-independent NHEJ may incorporate more nucleotides compared with DNA-PKcs-dependent NHEJ during DNA DSBs repair. Other characteristics of the mutation spectra in the brain of irradiated mice were similar between *scid* and WT mice.

## Conclusions

X-ray-induced deletions are predominantly generated by NHEJ in the brain and spleen of irradiated mice. NHEJ proceeds in DNA-PKcs-dependent and DNA PKcs-independent manners. DNA-PKcs contributes to NHEJ in the brain while it is dispensable in the spleen. This study suggests the organ specificity of the roles of DNA PKcs in deletion induction and raises a question of how NHEJ proceeds in the absence of DNA PKcs in mammalian organs.

## Supplementary information

**Additional file 1: Table S1.** Spi^−^ mutant frequency in the brain of X-ray-irradiated mice. **Table S2.** Spi^−^ mutant frequency in the spleen of X-ray-irradiated mice.

## Data Availability

All data generated or analysed during this study are included in this published article and its Supplementary Table 1 and 2.

## Abbreviations

DNA-PK: DNA-dependent protein kinase
DNA PKcs: The catalytic subunit of DNA PK
scid: Severe combined immunodeficient
WT: Wild type
MFs: Mutant frequencies
NHEJ: Non-homologous end-joining
DSBs: Double-strand breaks
IR: Ionizing radiation
Spi: Sensitive to P2 interference
SD: Standard deviation
bp: Base pair
APLF: Apratoxin and PNKP-like factor
FEN1: Flap endonuclease
DNA 2: DNA replication helicase/nuclease 2
ESTR: Expanded simple tandem repeat
